# Large-Scale Study of Antibody Titer Decay following BNT162b2 mRNA Vaccine or SARS-CoV-2 Infection

**DOI:** 10.3390/vaccines10010064

**Published:** 2021-12-31

**Authors:** Ariel Israel, Yotam Shenhar, Ilan Green, Eugene Merzon, Avivit Golan-Cohen, Alejandro A. Schäffer, Eytan Ruppin, Shlomo Vinker, Eli Magen

**Affiliations:** 1 Leumit Research Institute & Department of Family Medicine, Leumit Health Services, Tel Aviv 6473817, Israel; yshenhar@leumit.co.il (Y.S.); igreen@leumit.co.il (I.G.); emarzon@leumit.co.il (E.M.); agolanchoen@leumit.co.il (A.G.-C.); svinker@leumit.co.il (S.V.); allergologycom@gmail.com (E.M.); 2 Department of Family Medicine, Sackler Faculty of Medicine, Tel-Aviv University, Tel Aviv 69978, Israel; 3 Cancer Data Science Laboratory, National Cancer Institute, Bethesda, MD 20892, USA; alejandro.schaffer@nih.gov (A.A.S.); eytan.ruppin@nih.gov (E.R.); 4 Medicine C Department, Clinical Immunology and Allergy Division, Barzilai University Medical Center, Ben Gurion University of the Negev, Ashkelon 7830604, Israel

**Keywords:** antibody titer, BNT162b2 mRNA vaccine, SARS-CoV-2 infection

## Abstract

Immune protection following either vaccination or infection with SARS-CoV-2 is thought to decrease over time. We designed a retrospective study, conducted at Leumit Health Services in Israel, to determine the kinetics of SARS-CoV-2 IgG antibodies following administration of two doses of BNT162b2 vaccine, or SARS-CoV-2 infection in unvaccinated individuals. Antibody titers were measured between 31 January 2021, and 31 July 2021 in two mutually exclusive groups: (i) vaccinated individuals who received two doses of BNT162b2 vaccine and had no history of previous infection with COVID-19 and (ii) SARS-CoV-2 convalescents who had not received the vaccine. A total of 2653 individuals fully vaccinated by two doses of vaccine during the study period and 4361 convalescent patients were included. Higher SARS-CoV-2 IgG antibody titers were observed in vaccinated individuals (median 1581 AU/mL IQR [533.8–5644.6]) after the second vaccination than in convalescent individuals (median 355.3 AU/mL IQR [141.2–998.7]; *p* < 0.001). In vaccinated subjects, antibody titers decreased by up to 38% each subsequent month while in convalescents they decreased by less than 5% per month. Six months after BNT162b2 vaccination 16.1% subjects had antibody levels below the seropositivity threshold of <50 AU/mL, while only 10.8% of convalescent patients were below <50 AU/mL threshold after 9 months from SARS-CoV-2 infection. This study demonstrates individuals who received the Pfizer-BioNTech mRNA vaccine have different kinetics of antibody levels compared to patients who had been infected with the SARS-CoV-2 virus, with higher initial levels but a much faster exponential decrease in the first group.

## 1. Introduction 

Immunity to severe acute respiratory syndrome coronavirus 2 (SARS-CoV-2) has been induced either through SARS-CoV-2 infection or vaccination and induces protection against reinfection or decreases the risk of clinically significant consequences [1]. While one large study estimated that convalesced seropositive individuals have approximately 90% protection from SARS-CoV-2 reinfection, the effectiveness of vaccination has been reported as 50–95% [2,3]. Nevertheless, both the memory B cell humoral response and spike-specific CD4^+^ cellular immune responses to SARS-CoV-2 diminish over time [4,5]. Therefore, there is great concern regarding the weakened SARS-CoV-2 immune protection both in the vaccinated and convalescent populations [6]. 

Israel was among the first countries to initiate a large-scale vaccination campaign, on 20 December 2020, and quickly immunized a high proportion of the adult population, achieving early control over the spread of the virus [7]. More than five million Israelis (out of 9.3 million) were fully vaccinated with two doses of the Pfizer-BioNTech vaccine as of 26 May 2021 [8]. However, in summer 2021, there was a resurgence of SARS-CoV-2 cases in Israel. It is important to understand to what extent this resurgence is due to the high infectiousness of the delta variant [9], lower protection of the vaccine against the delta or other variants as compared to the original strain [10,11], or decreasing levels of anti-SARS-CoV-2 antibodies against all strains in vaccinated individuals [12]. 

Here, tracing one of these key factors, we describe the results of a large-scale study measuring the decrease rate of antibodies following administration of two doses of BNT162b2 vaccine, or SARS-CoV-2 infection in unvaccinated individuals in Israel. We show that these two populations are different demographically and hence, our analyses treat the vaccinated and convalescent populations separately. We use multivariable regression that largely corrects for demographic and comorbidity differences. Even with this correction, the kinetics of antibody decline in the convalescent and vaccinated populations appear to differ substantially.

## 2. Methods

### 2.1. Study Subjects and Study Design

We conducted a population-based study among adult members of Leumit Health Services (LHS), a large nation-wide health maintenance organization (HMO) in Israel, which provides services to over 700,000 members. LHS has a comprehensive computerized database, continuously updated regarding subjects’ demographics, medical diagnoses, medical encounters, hospitalizations, and laboratory tests. The socio-economic status (SES) was defined according to a person’s home address. The Israeli Central Bureau of Statistics classifies all cities and settlements into 20 levels of SES. Demographic groups weres also defined according to the home address of the HMO member, and categorized into three groups: General population, Ultra-orthodox Jews and Arabs; the latter two groups are of interest because a large-scale epidemiology study showed that they had significantly higher rates of infection than the rest of the Israeli population [13].

All LHS members have similar health insurance coverage and similar access to healthcare services. During each physician visit, a diagnosis may be entered or updated according to the International Classification of Diseases 9th revision (ICD-9). The validity of chronic diagnoses in the registry has been previously examined and confirmed as high [14,15]. 

We extracted serology results and associated demographic and clinical data for members aged 18 or older, who underwent a SARS-CoV-2 serology test between 31 January 2021, and 31 July 2021, following either two vaccine injections, or documented COVID-19 infection. Patients who had had received a vaccine injection and had a documented COVID-19 infection were excluded from the study.

Baseline data from individuals included in the cohort were extracted as of 15 May 2021, including age. All the clinical diagnoses were based on ICD-9 codes. During each physician visit, a diagnosis is entered or updated according to the International Classification of Diseases 9th revision (ICD-9). We tested for the main medical conditions expected to affect the severity of COVID-19 infection or the serology count in adult population: diabetes mellitus, hypertension, asthma, chronic obstructive pulmonary disease, ischemic heart disease, presence of malignancy, and chronic kidney disease. 

### 2.2. SARS-CoV-2 Testing by Real-Time RT-PCR

Nasopharyngeal swabs were taken and examined for SARS-CoV-2 by real-time RT-PCR performed with internal positive and negative controls, according to World Health Organization guidelines. The Allplex 2019-nCoV assay (Seegene, Seoul, Korea) and COBAS SARS-CoV-2 6800/8800 assay (Roche Pharmaceuticals, Basel, Switzerland) were employed. 

### 2.3. SARS-CoV-2 IgG Testing

Serum samples were run on the SARS-CoV-2 IgG lab-based serology blood test on the Abbot Alinity^™^ i system following the manufacturer’s instructions. In this antibody CMIA test, the SARS-CoV-2 antigen-coated paramagnetic microparticles bind to the IgG antibodies that attach to the SARS-CoV-2 spike protein (SP) in patients’ serum and plasma sample and it requires a minimum of 100 μL of serum or plasma. The resulting chemiluminescence in relative light units following the addition of anti-human IgG-labeled in comparison with the IgG II calibrator/standard indicates the strength of the response, which reflects the quantity of IgG to SP. IgG antibody levels measured by this test below 50 AU/mL are considered nonprotective. In internal testing, the Abbott Alinity^™^ system showed reliable results with 99.6% specificity and 100% sensitivity for COVID-19 patients tested 14 days after symptoms began [16]. The Abbott assay has been validated externally [17] with excellent sensitivity and specificity. Qualitative results and index values reported by the system were used in analyses.

### 2.4. Statistical Analyses

Standard descriptive statistics were used to present the demographic characteristics of patients included in this study and their measured antibody levels. Differences in demographic and clinical characteristics between groups were analyzed using the independent samples *t*-test, Mann-Whitney U test, and Fisher’s exact test for normally, non-normally distributed continuous, and categorical variables, respectively. Categorical data are shown in counts and percentages. Data on continuous normally distributed variables are represented by the mean and standard deviation. Non-normal variables are represented with the median and interquartile range. Linear regression models were fit to quantify the association between time since the second vaccination in vaccinated individuals or time since the first positive PCR in convalescents, and the logarithm of antibody levels. When converted to the logarithmic scale, zero values were replaced by one. For convenience, regression coefficients are displayed in figures and tables in the natural scale (after exponentiation). Multivariable regression models were also fit to measure the residual effect of time after adjusting the effect associated with age by category (18–59 years, more than 60), sex, demographic group, SES, comorbidity factors, and disease severity (presence of symptoms and admission to hospital during disease in convalescent patients). We performed the regression using time elapsed either as a continuous variable (which assumes linear effect), or as a binned categorical variable using 30-day intervals. Given that antibody titers appeared to increase in the initial 3 months after disease in convalescent individuals, we used the 90 first days as reference for the convalescent group.

### 2.5. Software

All statistical analyses were conducted using R software version 4.0.3 (R Foundation).

## 3. Results

During the study period, serology assays to quantify SARS-CoV-2 levels were performed for 2653 vaccinated individuals who never had a positive SARS-CoV-2 PCR test or serology test in the past, and 4361 patients recovering from SARS-CoV-2 and who had not been vaccinated at various times after the vaccination or infection. 

Table 1 describes the demographic characteristics and serology results for tested individuals in the vaccinated population and the COVID-19 convalescent individuals, according to the time that has elapsed until the serology test. The convalescent population was younger (41.99 ± 16.09 years) than the vaccinated population (56.45 ± 15.87 years) and was characterized by lower socioeconomic status (SES) and higher proportions of Ultra-orthodox and Arab subjects (Table 1). The mean period since the SARS-CoV-2 IgG lab-based serology test after the 2nd dose of vaccination was 101 ± 66 days, while since the first positive PCR in convalescents was 151 ± 82 days.

Table 2 and Table 3 display SARS-CoV-2 IgG antibody titers measured in vaccinated and convalescent individuals, in intervals spaced 30 days apart, since second vaccination (for the vaccinated) or first positive PCR (for convalescents). Lab-based serology is available for up to six months following vaccination for the vaccinated and up to nine months for convalescent patients. The age distribution of the patients for which serology was tested varies slightly throughout the follow-up, so it is indicated in Table 2 and Table 3. 

**Figure 1:**
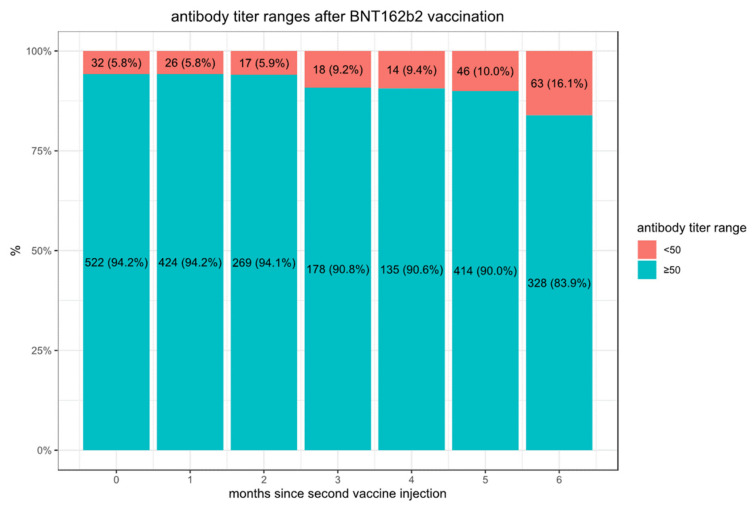
Proportions of doubly vaccinated individuals with antibody titers below the protective threshold of 50.

**Figure 2:**
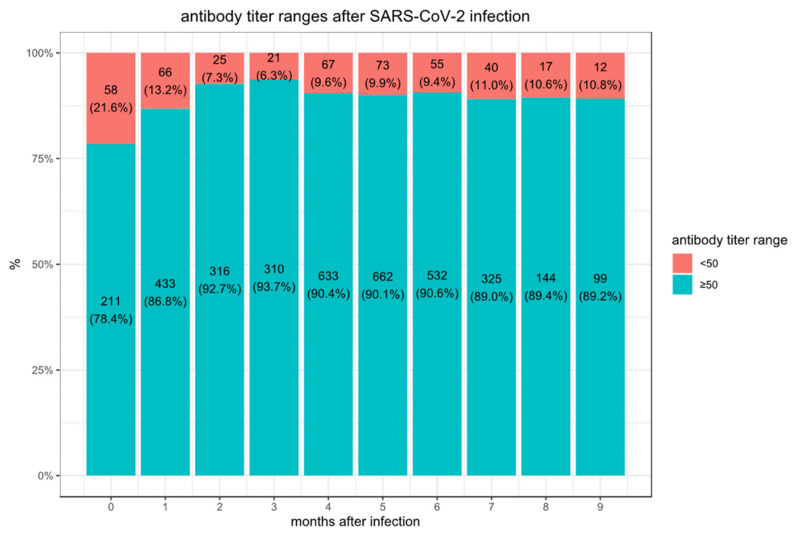
Proportions of convalescent individuals with antibody titers below the protective threshold of 50.

We observe considerably higher titers in the first month following the second vaccination (median 9913, IQR [3650–18,733]) than in convalescent patients after SARS-CoV-2 infection (median 490, IQR [109–1869] in the first month). In the convalescent subjects, the maximal mean antibody response was observed at 3 months after the documented COVID-19 infection, then the mean SARS-CoV-2 IgG antibody titer decreases slightly each subsequent month from the highest mean antibody response. The vaccination with the BNT162b2 vaccine-elicited much higher antibody titers at three months compared to the titers collected in serum from convalescent patients. However, in these vaccinated individuals who never had a positive PCR test, the mean SARS-CoV-2 IgG antibody titer decreased by approximately 40% each subsequent month from the highest mean antibody response. Consequently, we observed in BNT162b2 vaccinated subjects a worrisome decline in the proportion of people whose antibody levels are below the seropositivity threshold of <50 AU/mL (considered non-protective) from 5.8% in the first 3 months, to 16.1% after 6 months (Figure 1) while only 10.8% of convalescent patients are below the 50 AU/mL threshold after 9 months (Figure 2).

**Figure 3:**
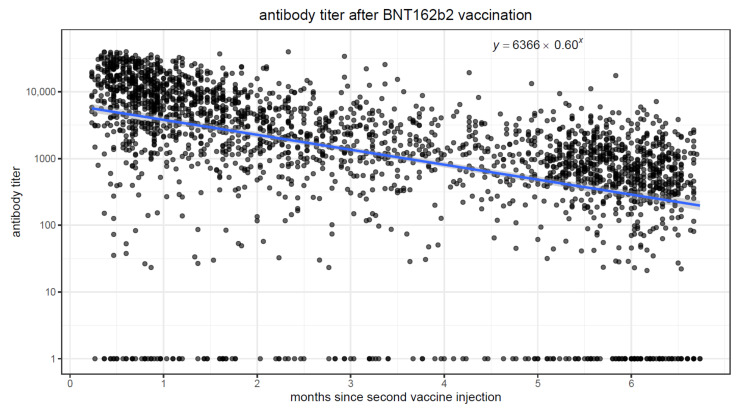
Scatter plot of time since second vaccination on the x-axis and antibody titer on the y-axis.

**Figure 4:**
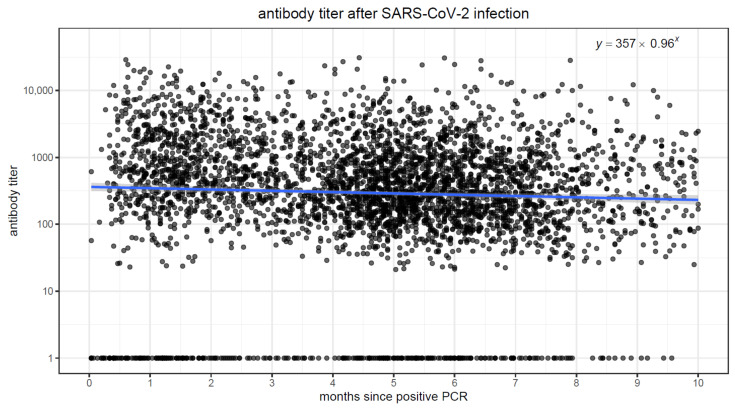
Scatterplot of time since infection on the x-axis and antibody titer on the y-axis.

We fit linear regression models to quantify the association between elapsed time and antibody levels, in both vaccinated and convalescent individuals. In both populations, there was a strong association (*p* < 0.001) between elapsing time and antibody titers. Figure 3 and Figure 4 display scatter plots with antibody titers plotted against elapsed time. In the vaccinated population, we observe higher initial antibody titers (intercept of 6366 at time zero), but the titers quickly drop, decreasing by approximately 40% in each passing month. Conversely, in the convalescent population, initial titers are lower (intercept of 357 at time zero), but the titers decrease much more slowly, by ~4% every month.

To adjust for the possible effects of age, sex, demographic group, SES in addition to time since the second vaccination or since the first positive PCR in convalescents on antibody levels, we performed multivariable regression models. Table 4 displays the regression coefficients on the vaccinated and convalescent cohorts using the time elapsed since vaccination or disease as continuous variables. In both populations, there was a strong association (*p* < 0.001) between elapsed time and antibody titers: each month was associated with a mean decay factor of 0.623 [95% CI 0.599–0.649] in vaccinated patients, while for convalescent patients the decrease was only by a factor of 0.960 [95% CI 0.939–0.982]. Among the vaccinated, antibody titers decreased with older age (factor 0.790 [95% CI 0.644–0.969] for age ≥ 60), chronic renal disease (factor 0.200 [95% CI 0.143–0.281]), underweight (factor 0.359 [95% CI 0.144–0.893] for BMI < 18.5), solid malignancy (factor 0.642 [95% CI 0.494–0.834]), COPD (factor 0.643 [95% CI 0.479–0.863]), patients with diabetes mellitus (factor 0.720 [95% CI 0.579–0.894]) and hypertension (factor 0.786 [95% CI 0.639–0.966]); they were increased in females (factor 1.243 [95% CI 1.035–1.492]) and in Arab and Jewish Ultra-orthodox subjects. In the convalescent, antibody titers were higher for symptomatic patients (factor 1.811 [95% CI 1.531–2.142]), those who had been admitted to the hospital (factor 3.323 [95% CI 2.217–4.980]) and those with risk factors for severe disease: older age (factor 1.546 [95% CI 1.269–1.884] for age ≥ 60), obesity (factor 1.839 [95% CI 1.166–2.899] for BMI > 35), diabetes mellitus (factor 1.354 [95% CI 1.093–1.678]), hypertension (factor 1.254 [95% CI 1.036–1.518]) and chronic renal disease (factor 1.965 [95% CI 1.134–3.407]).

Table 5 displays the regression coefficients in multivariable regression models featuring the time elapsed since vaccination or disease as binned 30 days intervals. Compared to the models displayed in Table 3, these models do not assume a linear decay of antibody levels. Nevertheless, we observe again that antibody levels decrease faster with time in vaccinated individuals compared to convalescent.

As shown in Table 2 and Table 3, the populations of convalescent and vaccinated individuals have different demographic characteristics, with the most obvious difference being the age distribution. We have used multivariable regression to attempt to adjust for differences in age, demographics, and comorbidities in Table 4 and Table 5. Because higher age is a risk factor for COVID-19, we also performed a subgroup analysis on individuals aged ≥ 60 in both convalescent (*n* = 712) and vaccinated (*n* = 1268) cohorts. In this subgroup analysis, the two groups are quite similar in age (mean age = 67.8 ± 6.5 for convalescents, and 69.2 ± 6.5 in vaccinated). Table 6 shows the regression coefficients in this subgroup. For vaccinated individuals aged ≥ 60 the mean decay factor was 0.619 indicating a decrease of about 38% per month; whereas for convalescent individuals aged ≥ 60, the mean decay factor was 0.889, indicating a decrease of about 12% per month. The decline of antibody titers was faster in convalescent individuals aged ≥ 60 than for younger convalescent individuals, and even among individuals aged ≥ 60, we observe a substantial difference in the rate of decay between convalescent and vaccinated individuals.

## 4. Discussion

In this large population of individuals tested for SARS-CoV-2 antibody titer following either vaccination or documented COVID-19 infection, we correlated antibody titers to elapsed time since exposure to vaccine or virus. Among never infected individuals who received the Pfizer-BioNTech mRNA vaccine, we found higher initial antibody levels followed by a faster decline compared to patients who had been infected with the SARS-CoV-2 virus. Consequently, the proportion of vaccinated individuals whose antibody levels drop below the threshold (50 AU/mL) thought to be protective increases substantially by the fifth month, while an antibody level below the protective threshold is uncommon in convalescent individuals.

### 4.1. Study Strengths

The strength of our study is that it provides antibody information in a large cohort of both vaccinated individuals and patients recovering from SARS-CoV-2. It shows that the declining slope of antibodies in vaccinated individuals is much steeper than in convalescent individuals. Importantly, the antibody measurements in the two populations were done in the same laboratory facility and with the same kits. The two populations have different demographic characteristics and, therefore, we analyzed the two cohorts separately. Yet, early in the vaccination phase, the most common way to assess whether the vaccination was effective was to compare antibody levels in newly vaccinated individuals to antibody levels in convalescent individuals (see the Section 4.3 and Section 4.4 below). To make this comparison possible, we used multivariable regression in order to correct for underlying differences. Other studies reported on the persistence of the humoral response in vaccinated subjects, but the follow-up time was usually below 3 months [18,19,20].

### 4.2. Study Limitations

This study has several limitations. First, given the observational design, there is potential for unmeasured confounding factors. In particular, participants in this study were individuals who elected to have a serology test for SARS-CoV-2 during the study period, many of them as part of a survey. Individuals may have variable reasons for accepting or refusing the offered serology test, which may have affected the results of this survey. The two groups varied in their age distribution and demographic characteristics, and we performed multivariable regression modeling to adjust statistically for factors that may be associated with antibody production. Nevertheless, additional, unidentified factors could have affected the results.

### 4.3. Related Work

Real world data about the time evolution of SARS-CoV-2 antibodies after vaccinations against SARS-CoV-2 and after the COVID-19 infection are limited. It had been shown previously in small-scale studies that the initial antibody levels post-vaccination are much higher than post infection [21,22], as confirmed in our much larger data set. Several studies reported on the humoral response following the BNT162b2 mRNA COVID-19 vaccination and found that SARS-CoV-2 antibody titers up to 6 months after infection decline more slowly in infected individuals [23,24,25]. Antibody levels post-infection tend to decline more steeply in individuals who had milder disease [26]. Three recent studies reported that antibodies could be detected up to 11 months after infection and provided evidence that that these antibodies originated in memory B cells [19,20,27]. Generation of SARS-CoV-2 memory B cells [28,29,30] is likely necessary for long-term protection in vaccinated individuals since that is the mechanism by which most anti-viral vaccines work [31].

Convalescent individuals may have a more diverse pool of memory B cells against SARS-CoV-2 than vaccinated individuals who were never infected [22,27]. Individuals who have recovered from COVID-19 have a significantly lower risk of SARS-CoV-2 reinfection. A recent 12-month longitudinal study showed that among convalescent COVID-19 plasma donors, the positive rate of IgG antibody against the SARS-CoV-2 receptor binding domain (RBD) in the spike protein exceeded 70% for 12 months post-diagnosis [32]. In our study, we show in contrast that following vaccination, the levels of anti-SARS-CoV-2 antibodies decrease rapidly, indicating that BMPCs may be lacking either in number or diversity and therefore anti-SARS-CoV-2 humoral immunity might be transient [33,34]. After infection, SARS-CoV-2 proteins and nucleic acids could remain in the gut for at least two months, boosting the continued antibody evolution in germinal centers, preferring epitopes overlapping with the ACE2-binding site on the RBD [35]. 

### 4.4. Importance of Antibodies in Protection against COVID-19

The BNT162b2 mRNA COVID-19 vaccine stimulates production of antibodies to several SARS-CoV-2 proteins, not just the spike protein, suggesting that the vaccine provides short-term protection against variant strains [18,19]; protection against variant strains has also been shown for the Moderna mRNA-1273 vaccine [36,37]. Combining these results with ours suggests that the increase in breakthrough infections in fully vaccinated individuals is due at least in part to declining levels of antibodies and not solely due to the variant strains of SARS-CoV-2. In a recent single-center, prospective, cross-sectional cohort study, children’s immunity was found to decline 4 months after the COVID-19 infection [20]. Lyer et al. reported that anti-S-protein antibodies reserved neutralizing abilities and persevered for up to 75 days after SARS-CoV-2 infection in >95% of patients [38]. Gudbjartsson et al. showed that SARS-CoV-2 IgG levels do not wane up to 4 months after infection [39]. 

Antibodies are not the only possible mechanism of immune protection and it has recently been shown that the BNT162b2 mRNA vaccine may elicit CD4^+^ and CD8^+^ T cell responses against SARS-CoV-2, [40]. However, it remains unclear whether the T cell responses are sufficient to protect against infection and if so, for how long. Effective humoral and cellular immune responses were observed a week after the booster dose, with the negligible immune response between the first and second doses [40]. Large-scale studies of long-term T cell responses to SARS-CoV-2 vaccination have been lacking [41].

It is widely accepted that neutralizing serum antibodies protect against SARS-CoV-2 in both nonhuman primates animal models and in humans [35,42] and hence measuring antibody levels as we did is useful to predict protection against infection. One limitation of our data is that the assay used in our study does not specially measure neutralizing antibodies; nevertheless, a high correlation was observed between a surrogate virus neutralization assay and other assays such as the Roche Elecsys anti-S pan-Ig assay [21,43]. 

## 5. Conclusions

Remarkably, after BNT162b2 mRNA vaccination, we observed higher SARS-CoV-2 antibody titers in the convalescent individuals aged ≥60 years, while in the vaccinated population higher SARS-CoV-2 antibody titers were seen in younger patients. Clinically, in a recent study performed in our health organization among individuals who had received two doses of the BNT162b2 vaccine, we observed that the rate of SARS-CoV-2 infection among patients who have received their second vaccine dose increased significantly for each 30 days elapsed after the initial 90 days post-second dose; the increase was significant for all age groups [12]. The decrease of SARS-CoV-2 IgG antibodies observed in the present study provides one explanation for the increased infection rate with increased time elapsed post-vaccination. Our observations call for replication in other populations to further correlate of protection against SARS-CoV-2 reinfection and/or COVID-19 disease and the duration of antibody-mediated protection.

## Figures and Tables

**Table 1:** The demographic characteristics of tested individuals in the vaccinated and convalescent population.

		Vaccinated	Convalescent
*N*		2653	4361
Age (in years)	mean (SD)	56.45 (15.87)	41.99 (16.09)
Age group *n* (%)	18–59 years	1296 (48.9%)	3663 (84.0%)
	≥60 years	1357 (51.1%)	698 (16.0%)
Sex, *n* (%)	Female	1604 (60.5%)	2728 (62.6%)
	Male	1049 (39.5%)	1633 (37.4%)
Demographic group, *n* (%)	Arab	248 (10.9%)	615 (14.1%)
	General (mostly Jewish)	1633 (71.9%)	1959 (44.9%)
	Jewish Ultra-orthodox	389 (17.1%)	1787 (41.0%)
SES, mean (SD)		9.88 (3.70)	7.57 (3.55)
	*missing*	179 (7.24%)	253 (6.16%)
Body mass index (BMI)	mean (SD)	27.79 (5.26)	27.20 (5.74)
	*missing*	35 (1.34%)	92 (2.16%)
BMI category, *n* (%)	<18.5 Underweight	46 (1.8%)	135 (3.2%)
	18.5–25 Normal	780 (29.8%)	1468 (34.4%)
	25–30 Overweight	994 (38.0%)	1470 (34.5%)
	30–35 Obese I	550 (21.0%)	781 (18.3%)
	35–40 Obese II	195 (7.5%)	304 (7.1%)
	40–Obese III	51 (1.9%)	105 (2.5%)
	*missing*	37 (1.4%)	98 (2.2%)
co-morbidities, *n* (%)	diabetes mellitus	659 (24.8%)	493 (11.3%)
	hypertension	1140 (43.0%)	808 (18.5%)
	asthma	299 (11.3%)	409 (9.4%)
	COPD	265 (10.0%)	137 (3.1%)
	ischemic heart disease	325 (12.3%)	183 (4.2%)
	solid tumor	342 (12.9%)	172 (3.9%)
	chronic renal disease	199 (7.5%)	58 (1.3%)
Time (in days) since… mean (SD)	2nd vaccination	101.35 (65.73)	-
	first positive PCR	-	151.17 (82.32)
Time (in days) since vaccination or positive PCR by 30 days intervals, *n* (%)	0–29	556 (21.0%)	269 (6.2%)
	30–59	456 (17.2%)	499 (11.4%)
	60–89	289 (10.9%)	341 (7.8%)
	90–119	200 (7.5%)	331 (7.6%)
	120–149	170 (6.4%)	700 (16.1%)
	150–179	542 (20.4%)	735 (16.9%)
	180–209	440 (16.6%)	587 (13.5%)
	210–239	-	365 (8.4%)
	240–269	-	161 (3.7%)
	270–	-	373 (8.6%)

SES: socio-economic status; BMI: body mass index; COPD: chronic obstructive pulmonary disease.

**Table 2:** Serology results of vaccinated individuals by 30 days intervals since second vaccination.

Time Since Second Vaccine Injection (in Days)		0–29	30–59	60–89	90–119	120–149	150–179	180–
*N*		556	456	289	200	170	542	440
Age (in years)		53.76 (16.90)	55.41 (16.66)	55.64 (16.52)	53.31 (16.75)	53.29 (16.29)	56.34 (13.76)	64.23 (12.30)
Sex, *n* (%)	Female	318 (57.2%)	273 (59.9%)	173 (59.9%)	129 (64.5%)	104 (61.2%)	358 (66.1%)	249 (56.6%)
	Male	238 (42.8%)	183 (40.1%)	116 (40.1%)	71 (35.5%)	66 (38.8%)	184 (33.9%)	191 (43.4%)
SARS-CoV-2 IgG antibody level	mean (SD)	12,153 (9875)	6848 (6340)	3476 (4582)	2383 (3266)	1552 (2103)	1122 (1431)	765 (948)
	median [IQR]	9913 [3650–18,733]	5106 [2109–9601]	2159 [1039–4169]	1323 [549–3126]	1071 [471–1901]	764 [385–1343]	447 [205–966]

**Table 3:** Serology results of convalescent patients by 30 days intervals since second first positive PCR test.

Time Since First Positive PCR (in Days)		0–29	30–59	60–89	90–119	120–149	150–179	180–209	210–239	240–269	270–
*N*		269	499	341	331	700	735	587	365	161	373
Age (in years)		39.96 (14.78)	41.10 (14.95)	45.07 (16.53)	43.52 (16.32)	42.90 (16.23)	40.95 (16.13)	41.39 (16.14)	40.59 (15.32)	41.75 (17.42)	43.18 (17.10)
Sex, *n* (%)	Female	157 (58.4%)	326 (65.3%)	234 (68.6%)	219 (66.2%)	400 (57.1%)	461 (62.7%)	371 (63.2%)	230 (63.0%)	112 (69.6%)	218 (58.4%)
	Male	112 (41.6%)	173 (34.7%)	107 (31.4%)	112 (33.8%)	300 (42.9%)	274 (37.3%)	216 (36.8%)	135 (37.0%)	49 (30.4%)	155 (41.6%)
SARS-CoV-2 IgG antibody level	mean (SD)	1914 (3870)	1739 (2972)	1552 (2522)	1195 (2406)	1079 (2556)	860 (1962)	904 (2384)	850 (2104)	901 (1739)	731 (1280)
	median [IQR]	490 [109–1869]	586 [212–1908]	538 [247–1723]	377 [165–1080]	329 [140–886]	312 [138–8301]	278 [125–751]	278 [105–727]	351 [124–919]	314 [116–783]

**Table 4:** Linear regression models of SARS-CoV-2 IgG antibody titer assuming linear decay with time. Regression coefficients were obtained by fitting a multivariable linear regression model for the log of antibody titer. They are displayed after exponentiation; they are therefore multiplicative: the expected antibody titer equals the intercept multiplied by each factor. Significant *p*-values are shown in **bold** font.

		Vaccinated			Convalescent		
		Factor	95% CI	*p*	Factor	95% CI	*p*
(Intercept)		10,598	[4889–22,975]	**<0.001**	234	[143–384]	**<0.001**
Each month since vaccination (for vaccinated)		0.623	[0.599–0.649]	**<0.001**			
Each month since first positive (for convalescent)					0.960	[0.939–0.982]	**<0.001**
was symptomatic (for convalescent)					1.811	[1.531–2.142]	**<0.001**
was hospitalized (for convalescent)					3.323	[2.217–4.980]	**<0.001**
Age	≥60 (vs. <60)	0.790	[0.644–0.969]	**0.024**	1.546	[1.269–1.884]	**<0.001**
Sex	Female (vs. Male)	1.243	[1.035–1.492]	**0.020**	0.923	[0.812–1.048]	0.215
Socio-economic status (SES)		0.995	[0.966–1.024]	0.723	0.995	[0.973–1.018]	0.662
Demographic grp. (vs. general)	Arab	1.525	[1.101–2.113]	**0.011**	0.982	[0.789–1.222]	0.871
	Ultra-orthodox	1.436	[1.099–1.877]	**0.008**	1.261	[1.065–1.492]	**0.007**
Body Mass Index (BMI)	<18.5 Underweight	0.359	[0.144–0.893]	**0.028**	0.683	[0.404–1.157]	0.156
	18.5–25 Normal	0.757	[0.374–1.534]	0.440	0.880	[0.581–1.333]	0.547
	25–30 Overweight	0.774	[0.383–1.564]	0.475	1.143	[0.754–1.733]	0.529
	30–35 Obese	0.804	[0.393–1.645]	0.550	1.429	[0.930–2.194]	0.103
	≥35 Obese II+	0.647	[0.307–1.363]	0.252	1.839	[1.166–2.899]	**0.009**
Comorbidity	diabetes mellitus	0.720	[0.579–0.894]	**0.003**	1.354	[1.093–1.678]	**0.006**
	hypertension	0.786	[0.639–0.966]	**0.022**	1.254	[1.036–1.518]	**0.020**
	asthma	1.200	[0.911–1.582]	0.195	1.046	[0.848–1.290]	0.676
	COPD	0.643	[0.479–0.863]	**0.003**	0.798	[0.562–1.133]	0.207
	ischemic heart disease	0.869	[0.655–1.152]	0.328	1.322	[0.956–1.828]	0.091
	solid tumor	0.642	[0.494–0.834]	**0.001**	1.048	[0.760–1.444]	0.777
	chronic renal disease	0.200	[0.143–0.281]	**<0.001**	1.965	[1.134–3.407]	**0.016**

**Table 5:** Linear regression models of SARS-CoV-2 IgG antibody titer according to days intervals. Regression coefficients were obtained by fitting a multivariable linear regression model for the log of antibody titer. Comorbidity covariates, same as those displayed in Table 4, were included in the regression. Significant *p*-values are shown in **bold** font.

		Vaccinated			Convalescent		
		Factor	95% CI	*p*	Factor	95% CI	*p*
days since vaccination (for vaccinated)	0–29	1.000	ref.				
	30–60	0.645	[0.497–0.838]	**0.001**			
	60–90	0.328	[0.243–0.442]	**<0.001**			
	90–120	0.174	[0.124–0.245]	**<0.001**			
	120–150	0.131	[0.089–0.191]	**<0.001**			
	150–180	0.100	[0.077–0.129]	**<0.001**			
	180–	0.064	[0.048–0.084]	**<0.001**			
days since first positive (for convalescent)	0–89				1.000	ref.	
	90–120				0.964	[0.754–1.232]	0.770
	120–150				0.786	[0.650–0.950]	**0.013**
	150–180				0.773	[0.641–0.932]	**0.007**
	180–210				0.720	[0.589–0.880]	**0.001**
	210–240				0.736	[0.581–0.934]	**0.012**
	240–270				0.868	[0.622–1.210]	0.403
	270–				0.691	[0.544–0.878]	**0.002**
Age	≥60 (vs. <60)	0.804	[0.656–0.984]	**0.035**	1.581	[1.300–1.921]	**<0.001**
Sex	Female (vs. Male)	1.251	[1.047–1.495]	**0.014**	0.919	[0.811–1.041]	0.184
Socio-economic status (SES)		1.006	[0.979–1.033]	0.680	0.989	[0.967–1.010]	0.301
Demographic grp. (vs. general)	Arab	1.731	[1.266–2.368]	**0.001**	0.916	[0.744–1.128]	0.408
	Ultra-orthodox	1.527	[1.185–1.967]	**0.001**	1.186	[1.011–1.391]	**0.036**
Body Mass Index (BMI)	<18.5 Underweight	0.335	[0.135–0.831]	**0.018**	0.630	[0.374–1.061]	0.082
	18.5–25 Normal	0.680	[0.339–1.362]	0.276	0.829	[0.550–1.250]	0.371
	25–30 Overweight	0.704	[0.352–1.408]	0.321	1.072	[0.711–1.617]	0.739
	30–35 Obese	0.699	[0.345–1.416]	0.320	1.321	[0.864–2.018]	0.198
	≥35 Obese II+	0.591	[0.284–1.232]	0.161	1.762	[1.126–2.757]	**0.013**

**Table 6:** Linear regression models of SARS-CoV-2 IgG antibody titer assuming linear decay with time analyzing only individuals of age ≥ 60. Regression coefficients were obtained by fitting a multivariable linear regression model for the log of antibody titer. They are displayed after exponentiation; they are therefore multiplicative: the expected antibody titer equals the intercept multiplied by each factor. Significant *P*-values are shown in **bold** font.

		Vaccinated			Convalescent		
		Factor	95% CI	*p*	Factor	95% CI	*p*
(Intercept)		3907	[592–25,761]	**<0.001**	185	[39–888]	**<0.001**
Each month since vaccination (for vaccinated)		0.618	[0.585–0.653]	**<0.001**			
Each month since first positive (for convalescent)					0.889	[0.839–0.943]	**<0.001**
was symptomatic (for convalescent)					1.746	[1.183–2.576]	**<0.005**
was hospitalized (for convalescent)					2.947	[1.597–5.436]	**<0.001**
Sex	Female (vs. Male)	1.401	[1.067–1.841]	**0.015**	0.865	[0.628–1.191]	0.373
Socio-economic status (SES)		0.989	[0.950–1.030]	0.593	1.035	[0.980–1.884]	0.218
Demographic grp. (vs. general)	Arab	1.782	[0.989–3.212]	0.055	1.020	[0.579–1.797]	0.945
	Ultra-orthodox	1.475	[0.987–2.206]	0.058	1.352	[0.881–2.073]	0.167
Body Mass Index (BMI)	<18.5 Underweight	0.147	[0.017–1.312]	0.086	0.476	[0.040–5.713]	0.558
	18.5–25 Normal	1.662	[0.269–10.246]	0.584	2.735	[0.624–11.991]	0.182
	25–30 Overweight	1.558	[0.254–9.544]	0.631	2.850	[0.669–12.144]	0.156
	30–35 Obese	0.804	[0.275–10.502]	0.569	3.060	[0.708–13.226]	0.134
	≥35 Obese II+	0.647	[0.200–8.116]	0.797	5.173	[1.161–23.042]	**0.031**
Comorbidity	diabetes mellitus	0.747	[0.570–0.979]	**0.035**	1.213	[0.864–1.704]	0.265
	hypertension	0.782	[0.594–1.030]	0.080	1.084	[0.773–1.519]	0.640
	asthma	1.284	[0.874–1.887]	0.202	1.386	[0.868–2.213]	0.171
	COPD	0.723	[0.501–1.042]	0.082	0.790	[0.462–1.349]	0.387
	ischemic heart disease	0.821	[0.592–1.139]	0.238	1.207	[0.796–1.831]	0.376
	solid tumor	0.574	[0.420–0.783]	**<0.001**	0.867	[0.546–1.379]	0.547
	chronic renal disease	0.329	[0.217–0.498]	**<0.001**	2.776	[1.301–5.921]	**0.008**

## Data Availability

This study is based on real-world patient data, including demographics, comorbidity factors, that cannot be communicated due to patient privacy concerns.
